# Methods for analyzing observational longitudinal prognosis studies for rheumatic diseases: a review & worked example using a clinic-based cohort of juvenile dermatomyositis patients

**DOI:** 10.1186/s12969-017-0148-2

**Published:** 2017-03-29

**Authors:** Lily Siok Hoon Lim, Eleanor Pullenayegum, Rahim Moineddin, Dafna D Gladman, Earl D Silverman, Brian M Feldman

**Affiliations:** 1 Children’s Hospital Research Institute of Manitoba, University of Manitoba, Winnipeg, Canada; 2 Department of Pediatrics, University of Manitoba, Winnipeg, Canada; 3 Institute of Health Policy Management and Evaluation, University of Toronto, Toronto, Canada; 4 The Child Health Evaluative Sciences Program, SickKids Research Institute, Toronto, Canada; 5 Department of Family and Community Medicine, University of Toronto, Toronto, Canada; 6 Department of Medicine, Toronto Western Research Institute, University of Toronto, Toronto, Canada; 7 Centre for Prognosis Studies, University Health Network, Toronto Western Hospital, Toronto, Canada; 8 Division of Rheumatology, SickKids, Toronto, Canada; 9 Physiology and Experimental Medicine Program, SickKids Research Institute, Toronto, Canada; 10 Department of Pediatrics and Immunology, University of Toronto, Toronto, Canada; 11 The Dalla Lana School of Public Health, University of Toronto, Toronto, Canada

**Keywords:** Epidemiology, Longitudinal study, Childhood-onset dermatomyositis, Biostatistics

## Abstract

Most outcome studies of rheumatic diseases report outcomes ascertained on a single occasion. While single assessments are sufficient for terminal or irreversible outcomes, they may not be sufficiently informative if outcomes change or fluctuate over time. Consequently, longitudinal studies that measure non-terminal outcomes repeatedly afford a better understanding of disease evolution.

Longitudinal studies require special analytic methods. Newer longitudinal analytic methods have evolved tremendously to deal with common challenges in longitudinal observational studies. In recent years, an increasing number of studies have used longitudinal design. This review aims to help readers understand and apply the findings from longitudinal studies. Using a cohort of children with juvenile dermatomyositis (JDM), we illustrate how to study evolution of disease activity in JDM using longitudinal methods.

**Electronic supplementary material:**

The online version of this article (doi:10.1186/s12969-017-0148-2) contains supplementary material, which is available to authorized users.

## Background

Outcome studies in rheumatic diseases have generally focused on single-occasion outcome assessments, e.g., 5-year outcome. When the outcome of interest is not a terminal event (e.g., death), but a dynamic one (e.g., disease activity, functional status), a cross-sectional view is usually not the best way to look at the data. Two patients may have similar outcomes at a point in time but *how* they arrived at their outcomes may have been very different. To understand the disease course, information about how outcomes change over time is necessary. By measuring patients’ outcomes repeatedly (by definition, on ≥ 3 occasions), a longitudinal study provides information about the shape of outcome trajectory, e.g., whether the disease goes into remission, waxes and wanes or remains persistently active [1].

Longitudinal studies require special longitudinal statistical analysis. Although some of these methods have been available for many years, they are not commonly used in the literature. These complex methods are harder to understand and use. This paper aims to: 1) Provide a review of common methods used to analyze longitudinal trajectory data; and 2) Demonstrate how to interpret results from longitudinal trajectory analysis [2–4]. We will focus on application of these methods to a real-life clinic-based rheumatic disease cohort.

### Questions that can be addressed by a longitudinal study

A longitudinal study can answer questions about the sources of variability in observed outcomes. In studies where outcomes are assessed once, the differences in outcomes are usually attributed to differences between individuals [1]. The effects of within-individual differences cannot be differentiated from that of between-individual differences in cross-sectional studies. In contrast, by measuring the outcomes repeatedly over time– for each individual– the longitudinal design captures important prognostic information about within-individual differences and allows these effects to be distinguished from between-individual differences.

Prognostic factors that fluctuate or emerge later during the course of the disease can only be understood using the longitudinal design. Examples of prognostic factors that often vary over time may include a biomarker of disease activity, or alterations in treatment. Using a longitudinal study design, we can repeatedly and simultaneously measure both the time-varying prognostic factors and the outcome(s) of interest, allowing direct relationships to be established.

### Special considerations in analyzing the longitudinal study

Methods to analyze longitudinal data have been available since the 1960s. Traditional longitudinal analyses include repeated measures analysis of variances (RANOVA) [5] and multivariate analysis of variance (MANOVA) [6]. Newer methods of longitudinal analysis include the generalized estimating equation (GEE) [7], mixed effects regression model (MRM) [8, 9], latent class trajectory analyses (LCTA) [10, 11], joint modeling [12–14] and multi-state modeling [15]. For illustrating longitudinal disease trajectory in this review, we will focus on the first four of these newer models. In this section, we will also compare the traditional with the modern methods of longitudinal analysis.

In a longitudinal study, each individual contributes at least three observations (by definition). As observations originating from the same individual are less variable than those originating from different individuals, longitudinal analysis needs to account for this relationship [16]. If analyzed without consideration for within-individual correlations, the conclusion will be inaccurate. However, traditional methods like RANOVA have highly restrictive assumptions [5], such as the assumption that the correlation between two measurements is constant, i.e., the correlation between measurements is similar, whether the measurements have been two days or two years apart. In contrast, newer methods attempt to account for the fact that within-individual correlations likely vary over time [17] (Additional file 1: Appendix).

In a longitudinal study, patients may be followed for differing lengths of time, resulting in a different number of visits for each patient and different visit schedules among patients. While traditional methods require an equal number of visits and/or the same schedule of visits, newer methods can accommodate an unequal number of visits and irregular measurement schedules [17].

Missing data is inevitable in observational longitudinal studies. As patients are followed over long periods, there will be times when patients may leave a cohort and then return, or be lost to follow-up. Traditional longitudinal methods have a requirement of no missing data, which is impractical in longitudinal observational studies [18]. In contrast, newer methods are able to handle missing data with varying degrees of flexibility [7, 8, 19] (Additional file 1: Appendix).

We will now apply four of the longitudinal methods– GEE, MRM, LCTA and Joint Modelling– to illustrate the use of these methods in an observational cohort. We have chosen these four models, as they all provide a view of the shape of the longitudinal outcome trajectory and form the basic models from which more complex models can be developed.

### Juvenile dermatomyositis as a disease model for longitudinal analysis

We will use Juvenile Dermatomyositis (JDM) as a convenient disease model to show how longitudinal design and analysis can be used in rheumatic diseases. Multiple cross-sectional studies have determined that, when assessed 2-3 years after diagnosis, there are three disease course patterns in JDM: monocyclic, polycyclic or chronic [20–23]. Although previous studies have shown that a substantial proportion of patients have active disease many years after the diagnosis [24, 25], these studies could not differentiate patients with active disease throughout their entire disease course from those with a polycyclic course. A longitudinal study can help to clarify this question.

The study population was 95 JDM patients followed at The Hospital for Sick Children, Toronto, Canada. Information about this cohort has been previously reported [26, 27]. We used clinical data from the first four years of follow-up to demonstrate the application of longitudinal analytic methods. The frequency of patients’ visits was based on the severity of their disease, i.e., the visit schedule was irregular across the population. See Additional file 1: Appendix for the baseline characteristics of this cohort.

The primary outcome was the modified Disease Activity Score (DASm) [27, 28]. The skin component of modified DAS (SDAS) scores up to 4 points and the musculoskeletal component (MDAS) up to 7 points.

We used: 1) The DASm at diagnosis (bDAS) as an example of a time-invariant (unchanging) prognostic factor; and 2) The steroid dose (in mg/kg) or methotrexate use (yes or no) from visits before each DASm measurement as examples of time-varying (changing) prognostic factors. As we used bDAS as a predictor, we excluded the first visit DASm (bDAS) in this dataset. We tested 3 different lag times (of 3, 6 and 12 months) when the time-varying predictor (treatment) was measured (e.g., methotrexate use 3 months before, 6 months before or 12 months before DASm measurement).

### Questions and answers

#### Question 1: What is the disease activity course for a population of JDM patients?

The GEE, which determines the mean population disease activity trajectory, is frequently used to answer this kind of question [7]. The GEE calculates the average DASm of the whole population at each visit. These population averages are then joined to make a “trajectory” of DASm for the whole population over time. The GEE assumes that the measurement schedule is unrelated to the outcome, i.e., both sick and well patients are presumed to have the same visit schedule on average. As sicker patients were likely seen more frequently than those who were well, this assumption was probably not supported in our clinic-based cohort. However, there have been extensions of the GEE (the weighted GEE) that allow GEE to be used even when the measurement schedule is related to the outcome [29].

The GEE is the most popular method used for longitudinal analysis for several reasons [30]. With the GEE, the mean population response at each occasion is modelled as the result of only the prognostic factors of interest and not of previous responses or random effects (individual heterogeneity) [9]. If the researcher’s interest is in the average population prognosis, then the GEE provides a simple interpretation [31].

**Fig. 1:**
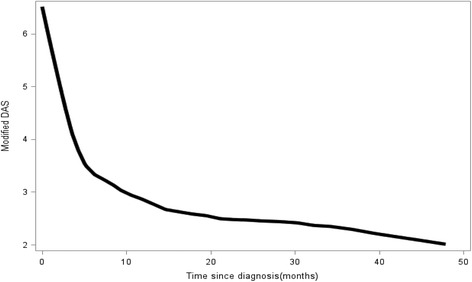
Population-averaged modified DAS trajectory in JDM patients (representation of GEE). Disease activity score, DAS. Total population = 95

We will briefly discuss the results when we used the GEE to analyze our study cohort. From the smoothed local regression plot of Fig. 1 [32], we can see that the average population DASm decreases rapidly and then plateaued to a lower degree of disease activity. This is clinically sensible as there are effective treatments for lowering disease activity in JDM, yet many patients remain chronically active.

In summary, the GEE is a good choice for analyzing studies where the overall population prognosis is the primary interest, e.g., when addressing questions about population trends or healthcare utilization.

#### Question 2a: How do we determine the disease activity course of an individual with JDM?

**Fig. 2:**
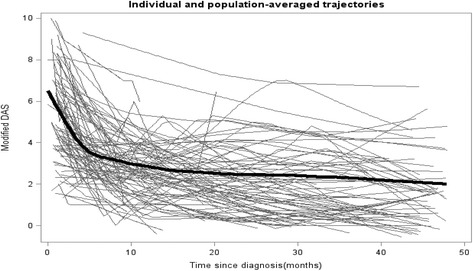
Plot of all JDM patients’ individual and the population-averaged modified DAS trajectories (representation of MRM). Disease activity score, DAS. Total population = 95

The best way to address this question is to use the MRM, a so-called subject-specific model, that allows for potential inference at the level of the individual [1, 33]. For a continuous outcome like DASm, the MRM also provides us with the average population trajectory. The smoothed plot (Fig. 2) shows the DASm trajectories of both the individual patients (gray lines) and the mean population trajectory (bold black line) [32]. As the outcome is continuous, fitting the MRM resulted in a similar longitudinal trajectory to that derived from the GEE. However, if the outcome is not continuous, calculating the mean population trajectory from the MRM is very complex.

In the MRM, each individual’s trajectory is different from the mean population trajectory because of “random effects.” Random effects are a combination of *unmeasured* prognostic factors, confounders, environmental factors and genetic factors, which account for heterogeneity within the population [31]. Any difference between an individual’s trajectory and the average population trajectory is the result of that individual’s random effects; this forms the basis for individual level inference from the MRM. Random effects may be applied at intercepts, which represent between-individual differences. When random effects are applied to slopes, this allows individuals’ time trend to deviate from the average population trajectory. Although the random effects are taken into account in modeling, the results reported are that of the mean population trajectory in this case.

Although the results of the MRM may appear much like the GEE in continuous outcomes models, the interpretations are very different. The MRM models the average of individuals’ trajectories (subject specific) but the GEE models the trajectory from DASm averages at each time point (population average). Practically, in the context of the continuous outcomes models, the forms and the results from the 2 models are very similar. The differences between MRM and GEE are obvious when the outcomes are non-continuous, e.g., binary or ordinal [9, 34–36]. Taking the example of a random intercepts (logistic) MRM, where the outcome could be disease remission (yes/no), and a time-invariant covariate could be calcinosis at baseline (yes/ no), the odds of remission would correspond to the effect of calcinosis plus an individual’s random effects. The effect of calcinosis therefore changes with different individuals. The odds ratios estimated by the logistic MRM are subject specific as these additionally adjust for heterogeneity between individuals. In the binary outcome GEE, the odds ratio corresponds to odds of an event among those with baseline calcinosis divided by the odds of an event in those without. As these are ratios of subpopulation risks, the GEE estimates are termed population average. From this, it should also be obvious that the results of non-continuous MRM will always be greater that than of non-continuous GEE due to inclusion of the random effects. The choice of model depends on the purposes of the study. If the mean prevalence of disease remission in a population over time predicted by baseline calcinosis is of interest, the GEE is suitable. If the investigators want to *study individuals’ risk factors for personal predictions*, then the MRM is the model of choice. As the estimates of the MRM are adjusted for random effects (unobserved individual characteristics), the results reflect the effect of baseline calcinosis status of an individual with a specific random effect (or individuals with the same random effect).

#### Question 2b: How do we predict the disease activity course of an individual with JDM?

**Fig. 3:**
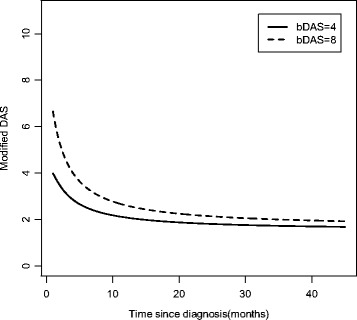
Effect of baseline (time-invariant) modified DAS on the modified DAS trajectory in JDM patients (MRM). Activity Score, DAS; Baseline modified DAS (bDAS)

i) Time-invariant prognostic factor
  The MRM can be used to determine the individual level predictive effect of a time-invariant prognostic factor, such as the baseline DASm (bDAS), on the trajectory of disease activity. From Fig. 3, we can see that the average trajectory for individuals with a higher bDAS had a faster initial improvement (up to month 10) followed by persistently higher activity, compared to the average trajectory of individuals with a lower bDAS (*p* < 0.0001). Baseline state therefore predicted the rate of change of the slope of the trajectory. We can therefore tell patients that their disease activity will likely improve within 1 year (with treatment). Those who are more active initially (higher bDAS) will likely see a relatively faster improvement in their symptoms compared to those who are less weak.

ii) Time-varying prognostic factor
  Using the MRM, we tested the individual level effects of time-varying prognostic factors, including corticosteroid and methotrexate exposures from 3, 6 and 12 months before each visit. In this case, the time-varying predictors tested did not influence the DASm trajectory. Had these time-varying predictors been significant, their effects would have been to shift the trajectory up or down, corresponding to slower or faster resolution of disease activity.
  In summary, if the primary interest of a study is to understand how covariates impact disease course, based on the *individual* patient’s prognosis trajectory, the MRM is the best choice. The MRM is also more suitable when there is significant heterogeneity in individuals’ disease courses in the population.

#### Question 3a: Are there different patterns (or subgroups) of disease activity course among individuals with JDM?

The models used in the preceding sections assumed that the shape of patient trajectories were homogenous, i.e., all patients follow the same disease course. If the investigator suspects that his study population contains heterogeneous patient trajectories, then latent class trajectory analysis (LCTA) (Additional file 1: Appendix) can be used to identify distinct subgroups with different disease trajectories (those with a clinically distinct prognosis) [10, 37].

**Fig. 4:**
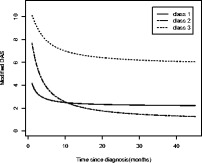
Latent classes of JDM disease activity trajectories (LCTA). Disease Activity Score, DAS. Class 1, *n* = 49; Class 2, *n* = 43; Class 3, *n* = 3

When we applied LCTA to our cohort, we found three distinct subclasses. Each of these three subclasses has a class-specific average population disease activity trajectory, derived using individuals’ trajectories (with the MRM as in question 2a). From Fig. 4, we can see that Class 2 patients have high disease activity at diagnosis, and then rapidly improve. Class 1 patients have moderate disease activity at diagnosis that improves gradually to low disease activity over time. Class 3 patients have high disease activity at diagnosis that improves very gradually. For this kind of model, we determine a probability of belonging in each of the three classes but for each individual, but the probability of any individual of belonging to each class varies. When classified according to the highest probability of class membership for each individual, 42% are in class 1, 55% in class 2 and 3% in class 3. None of the patients was classified into 2 classes with similar probabilities, i.e., was ambiguous in the class membership probability (see Additional file 1: Appendix).

The LCTA is very flexible (Additional file 1: Appendix): 1) More than one and more than one kind of outcome trajectory can be modelled at a time (see next section); and 2) Trajectories of different subclasses can take on different shapes (reflecting different patterns of outcome evolutions) [37–39].

#### Question 3b: What factors predict an individual’s membership in the different subgroups of disease activity?

The LCTA allows us to study the effects of time-invariant baseline factors, such as bDAS, in predicting membership in distinct subgroups. We use the bDAS to predict membership in the three subclasses. The higher the bDAS, the less likely is a patient to belong in class 1 (OR 0.11, 95% CI 0.02-0.63) or 2 (OR 0.27, 95% CI 0.05-1.41) compared to class 3. Furthermore, the higher the bDAS, the less likely is a patient to belong to class 1 (OR 0.41, 95% CI 0.25-0.68) compared to class 2. This means that those with highest bDAS are most likely to belong to class 3, with chronic high-grade disease activity. High bDAS patients are less likely to improve substantially or go into low disease activity states unlike those whose initial bDAS are low to moderate. This may appear different from the results of MRM where high bDAS predicted quicker resolution. In MRM, the whole population was studied together although there was significant heterogeneity within the population. LCTA allows us to group patients into more homogeneous subgroups and then more precisely clarify the effect of potential baseline membership predictors.

Time-varying covariates can also be studied in LCTA and can be formulated in different ways depending on the underlying question [40, 41]. As the computations are very complex and interpretations challenging, we chose to leave out time-varying covariates for this review.

In summary, if a researcher suspects that there are several prognostic subgroups within the study population, the LCTA can be used to identify these subpopulations. Baseline factors may identify patients’ memberships in different prognostic subpopulations, thus allowing more individualized management. Time-varying covariates can also be studied but interpretations are more complex (Additional file 1: Appendix).

#### Question 4a: What are the separate disease activity courses for the skin and musculoskeletal components of JDM and what is the relationship between these two disease components?

**Fig. 5:**
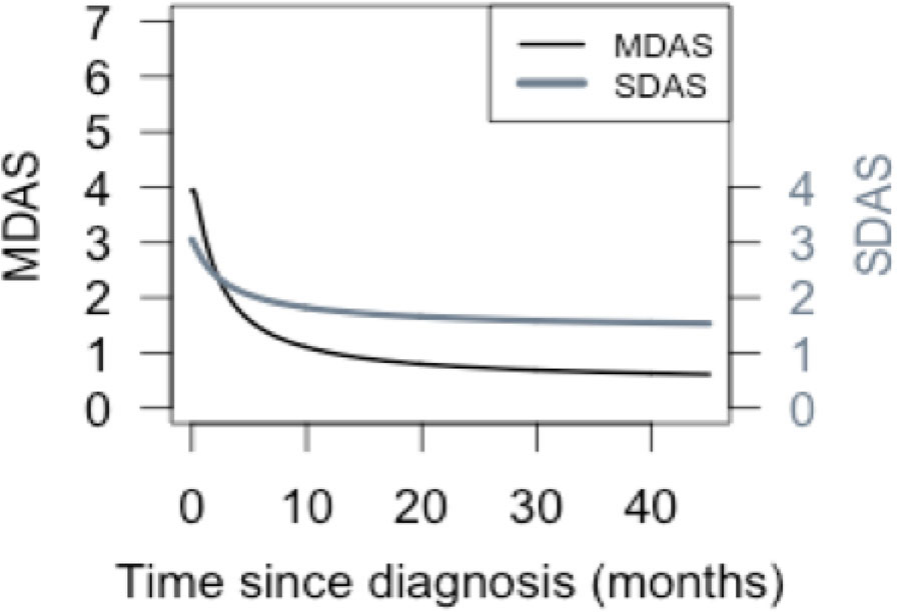
Musculoskeletal and skin disease activity trajectories in JDM (joint multivariate modeling). Disease Activity Scores, DAS; Musculoskeletal DAS, MDAS; Skin DAS, SDAS. MDAS and SDAS are components of the modified DAS. The left y axis (*black*) represented the MDAS (maximum score = 7) and the right y-axis (*grey*) represented the SDAS (maximum score = 4). The MDAS and SDAS curves are colour coded to match their respective axes

In this case, we have two potential outcomes: the modified skin (SDAS) and musculoskeletal (MDAS) components of JDM. We are interested in how the disease courses evolve over time and the correlation of disease activity in the two components of JDM over time. The best way to study how two different outcomes evolve simultaneously over time is a joint model as shown in Fig. 5 (Additional file 1: Appendix) [12–14, 42, 43]. The joint model refers to the concurrent modeling of two outcome trajectories (SDAS and MDAS) in one model. The basic model used here to model the trajectories is also the MRM.

The two disease components of JDM follow different trajectories. The MDAS improves more rapidly (within the first 10 months after diagnosis) to minimal activity. In contrast, the SDAS improves less rapidly and persists at a low activity level for almost 20 months (Fig. 5). The two disease components therefore follow discordant courses, as confirmed by the low correlation between the two trajectories (*r* = 0.32) [13]. This relationship between two or more outcome trajectories can only be studied using the joint model and not by using separate models for the individual components.

In our example above, both outcomes were treated as continuous outcomes. However, outcomes of different natures can be jointly modeled. We can model a continuous outcome (e.g., DASm), with a time-to-event outcome (e.g., calcinosis), a binary outcome (e.g., nailfold capillary abnormality) or a count (e.g., active joint count). Furthermore, as alluded to previously, more than one outcome can be jointly studied in the LCTA; in this case, members within the same subclass of joint outcomes would have similar trajectories or risk of events [38, 39, 44]. This way of examining outcomes more closely resembles the real world where several clinically relevant outcomes may be considered equally important in clinical decision making.

#### Question 4b: What factor(s) predicts the joint disease activity courses of the skin and musculoskeletal components of disease in an individual with JDM?

We evaluated the predictive effects of bDAS (time-invariant) on the joint MDAS and SDAS trajectories (Table 1). In this joint model, individuals with higher bDAS have a faster decline in the slope of the MDASm trajectory; but bDAS does not significantly affect the slope of the SDAS trajectory. Prior treatment with methotrexate or steroid (time-varying factors) does not significantly influence the MDAS and SDAS trajectories, perhaps because everyone received the same protocol of treatment. Thus, joint modeling can distinguish the prognostic effects of predictors for the major components of JDM disease activity.

**Table 1:** Predictors identified from the joint multivariate model of MDAS and SDAS

Predictor	Outcome	Predictor Estimate	Standard Error	*p*
bDAS	MDAS	0.0363	0.0316	0.25
	SDAS	0.0214	0.0342	0.53
Time^a^*bDAS	MDAS	1.0140	0.1321	**<0.0001**
	SDAS	0.0362	0.1261	0.77
Steroid^b^	MDAS	-0.0919	0.0753	0.22
	SDAS	0.1211	0.0688	0.08

*bDAS* Baseline DASm measurement; Time *bDAS denotes the crossing of a time term with bDAS

^a^The shapes of the MDAS and SDAS models are defined using 2 time terms each (fractional polynomials). MDAS and SDAS crossed with their common time form (*p* = –1,) of their respective fractional polynomials (see Additional file 1: Appendix)

^b^Steroid treatment from 3 months before each occasion of DASm measurement, i.e., it is a time-varying predictor. Significant results have been bolded

In summary, if a researcher is interested in more than one (or more than one kind of) outcome and wants to evaluate the relationship (i.e., correlation) among these outcomes over time, the joint model can be used. The joint model can be used in the LCTA context so that latent classes of the different outcomes of interest can be delineated. The joint model also allows direct comparisons of the differing effects of the same predictors on multiple outcomes simultaneously.

## Conclusions

In this paper, we have illustrated the use of longitudinal design and analysis in studying prognosis. Longitudinal studies are superior to cross-sectional studies as they use all available data, thereby giving a more complete view of patients’ outcomes.

We have presented four different ways of analyzing longitudinal observational data depending on the question(s) of interest. The GEE is best used if a population-level view is preferred and the visit schedule is not related to the outcome studied. This population view may be more relevant to health services researchers addressing population level questions, e.g., healthcare utilization. The MRM should be used if an individual level view is of interest. The LCTA should be used when the researcher wants to identify subgroups of patients within a heterogeneous cohort, with different outcome trajectories, and identify the factors predicting their membership in these subgroups. Joint modeling is best used to study the evolution and correlation among multiple outcome trajectories and the differing effects of predictors on simultaneous multiple outcome trajectories. We have summarized salient points about the four models in an overview table (Table 2). These four methods can also be combined with other methods, e.g., propensity scoring [45] and marginal structural modeling [46], to answer other kinds of questions (e.g., therapeutic) in a longitudinal study [47].

**Table 2:** Overview of the 4 modern longitudinal analytic methods

Model	Questions	Advantages	Disadvantages
GEE	What is the averaged outcome trajectory for the population? (Trajectory of averages)	Parameter estimates robust to misspecification of the covariance structure. Both time-invariant and time-varying predictors can be studied.	No individual level inference Assumes missing data to be missing completely at random (MCAR), which may not be true for many longitudinal studies.
MRM	What is the outcome trajectory of the individual? What is the average outcome trajectory for the population? (Average of trajectories)	Individual level inference possible with the incorporation of random effects. Both time-invariant and time-varying predictors can be studied. Assumes missing data to be missing at random (MAR), which is more likely in longitudinal studies.	Misspecification of covariance structure may bias parameter estimates^45^
LCTA^a^	Are there distinct subgroups within the study population? What are the trajectories of the identifiable subgroups within the population?	Objectively identifies latent distinct subgroups within a heterogenous population. Able to use time-invariant factors to predict group membership. Able to study effects of time-varying covariates in different ways (depending on question and underlying theoretical framework) Assumes data to be missing at random (MAR).	Complex and time-consuming computing procedures. Interpretation of time-varying covariates can be challenging depending on the formulation.
Joint Model^b^	What are the trajectories of (multiple) outcomes of interest? What is the correlation between the outcome trajectories of interest (i.e., are the trajectories concordant or discordant)?	Multiple outcome trajectories of disparate nature (e.g., continuous with binary, binary-poisson, continuous-survival) can be studied simultaneously. Objective determination of the longitudinal correlation of the trajectories. Joint model with time-to-dropout may be used as a means to adjust for data missing not at random (MNAR).	Modeling procedures can be complex with increasing number and kinds of outcomes modeled jointly.

^a^Usually modeled with MRM as the base model

^b^MRM may be used as the base model for continuous, binary and count data. Proportional hazard is used for time-to-event outcomes

While the methods presented in this paper have the potential to transform our understanding of prognosis, we acknowledge that these methods could be challenging to use without necessary expertise. We therefore recommend consulting with biostatisticians knowledgeable in these methods to help design and analyze longitudinal studies. Our review is not meant to be an exhaustive review of all available longitudinal analytic methods. For example, Markov multistate models can also be used to determine patients’ transition between disease states longitudinally [15]. In the interest of simplicity, we tested a minimum number and kind of predictors in these models. In practice, more predictors can be tested in these models, with a far greater confidence in predicting individuals’ disease trajectories.

The methods outlined in this paper will allow for a more complete understanding of longitudinal outcomes and a more precise understanding of the effects of predictors. Combination of these methods with molecular information holds great potential to transform clinical practice towards the ultimate goal of precision medicine.

## Additional file

Additional file 1: Appendix of longitudinal analysis methods for prognosis study in rheumatic diseases. (DOCX 1112 kb)

## Abbreviations

bDAS: Baseline disease activity score
DASm: Modified disease activity score
GEE: Generalized estimating equation
JDM: Juvenile dermatomyositis
LCTA: Latent class trajectory analysis
MANOVA: Multivariate analysis of variance
MDAS: Musculoskeletal disease activity score
MRM: Mixed effects regression model
RANOVA: Repeated measures analysis of variance
SDAS: Skin-disease activity score
