# Supplementary material for: Methods for analyzing observational longitudinal prognosis studies for rheumatic diseases: a review & worked example using a clinic-based cohort of juvenile dermatomyositis patients
Source: Pediatr Rheumatol Online J. 2017 Mar 29;15:18. doi: 10.1186/s12969-017-0148-2 (PMC5371187; doi:10.1186/s12969-017-0148-2)
Supplement: Additional file 1: — Appendix of longitudinal analysis methods for prognosis study in rheumatic diseases. (DOCX 1112 kb) [file 12969_2017_148_MOESM1_ESM.docx]

This supplementary document has been prepared to include further information about the study cohort used for illustration and to provide interested readers a more in-depth understanding of the technical aspects of newer longitudinal analytic methods.

**STUDY COHORT**

We used a cohort of Juvenile Dermatomyositis (JDM) patients followed at our centre for illustration of the application of modern longitudinal analytic method. Patients were diagnosed and followed between 1^st^ January 1991 and 31^st^ December 2010. Table A1 shows the baseline characteristics and follow-up information of this cohort of patients.

**Table A1: Baseline Characteristics and Follow-up Information of Study Patients**

| **Characteristics** | **n=95** |
| --- | --- |
| Gender: Males (%) | 33 (35) |
| Median age at diagnosis, years (25%- 75% percentile) | 7.8 (4.9- 12.1) |
| Time- period of diagnosis, n (%)  1991-1999  2000-2010 | 37 (39)  58 (62) |
| Median baseline DAS (modified) at first visit (25%- 75% percentile)* | 7 (6-9) |
| Median duration of follow-up, years (25%- 75% percentile) | 3.5 (2.4-3.8) |
| Median number of visits per patient (25%- 75% percentile) | 14 (10-16) |
| Total follow-up of the cohort, patient years | 285.64 |

* Disease Activity Score, DAS.

**DATA ANALYSIS**

We first examined the modified disease activity score (DASm) trajectories of all patients within this cohort (Fig 1) for possible trajectory shapes. From perusal of the individual trajectories, it was clear that the relationship of DASm with time was non-linear and did not clearly fit with any conventional low order polynomials.

**Figure A1: Plot of all JDM patients’ modified DAS trajectories**

*Disease Activity Score, DAS. This referred to the modified version (DASm).*

In order to model the evolution of the DASm trajectories more accurately, we tested various combinations of fractional polynomials [[1](#_ENREF_1), [2](#_ENREF_2)]. We tested first order (m=1) and then second order (m=2) fractional polynomials. For the second order fractional polynomials, we tested the powers (p) that have been suggested by Royston to fit most biomedical data, i.e. p= -2, -1, -0.5, 0, 0.5, 1, 2, 3. The DASm curve was best fitted by a 2^nd^ order fractional polynomial (m=2,p= (-2,-1)), with maximal gain [[1](#_ENREF_1)].

The analyses to be presented in the following sections were performed using SAS (Cary, North Carolina, USA) or R (http://www.r-project.org) where appropriate.

**LONGITUDINAL ANALYTIC METHODS**

**1) GENERALIZED ESTIMATING EQUATION (GEE)**

Currently, the GEE is probably the most commonly used analytic method for longitudinal studies[[3](#_ENREF_3), [4](#_ENREF_4)]. In the simplest form, this is like a linear regression, plus a correction factor for the within-person correlation of measurements. It is a marginal model, meaning it presents a population averaged view of the repeatedly measured outcomes at each time point.

How does the GEE answer our question about the modified disease activity score (DASm) trajectory in JDM patients? The GEE equation fits a trajectory connecting mean population DASm at each occasion of measurement, giving an overview of the population’s average DASm evolution over time. This has two implications. First, it is important to note that the marginal trajectory is a curve of averages (at the occasions of measurements), not an average of curves: we therefore cannot necessarily infer an individual’s DASm trajectory from this equation, especially if the outcome is non-continuous. Second, to accurately understand the population averaged DASm at each measurement, it is implied that there is a similar schedule of visits for the cohort; although some degree of irregularity can be tolerated. Furthermore, there is an assumption that this schedule of visits is not related to the outcome in any way. For cohorts where there are no regular schedules of assessments or where patients may visit more often if they are sicker (i.e. the visit schedule is informative), the GEE is not a good choice of analytic method[[5](#_ENREF_5)]. In such cases, an extension of the GEE, weighted GEE (WGEE), which uses inverse probability weightings of the visits, will be more appropriate[[6](#_ENREF_6)].

In the GEE, within-subject correlation is accounted for by including a covariance structure in the model. As GEE uses quasi-likelihood, this covariance matrix need not be completely accurate for the estimates to be consistent[[3](#_ENREF_3)]. Misspecifying the covariance matrix will still however negatively impact on the efficiency of the model.

The GEE does allow missing data. The assumption here is data are missing completely at random (MCAR)[[7](#_ENREF_7), [8](#_ENREF_8)]. This means that the missing data must be neither related to the missing outcome nor be predictable by covariates or the history of outcomes. An example of this would be a child who stops attending our JDM clinic as his family has relocated to another province for his father’s new job. This has nothing to do with his disease condition and is not predictable based on his intrinsic characteristics or his disease outcomes up to that point. The MCAR assumption may be difficult to fulfill in real life cohorts. It is important to explore the mechanisms of missing data as having missing data that are not MCAR will bias the inference derived from the GEE. If the interest is in population inference and there is evidence of data missing at random, the WGEE can be used instead as it tolerates missing at random (MAR) mechanism for missing data. MAR missing data are not related to the missing outcome but may be predictable from covariates (e.g. patient characteristics) or outcomes up to the point of attrition. An example of this would be the observation that JDM patients who live further away (patient characteristic) tend to not return for visits when they are better (history of outcomes up to attrition).

Both time-invariant and time-varying predictors can be evaluated using GEE. The time-invariant predictor associates with the intercept and predicts the slope of the trajectory. To interpret the meaning of a time-varying predictor in GEE however, one needs to consider where the source of variation is, whether it is between or within- individual. In this case, as most subjects were started on a standard dose of prednisone (i.e. minimal between-individual variation), most of the variation occurred within the individual over time. Conversely, if a time-varying covariate varies more between individuals and fluctuates less within an individual, most of the variation would be between-individual and such a variable would predict differences between individuals. One can easily see that if a time-varying covariate varies between individuals and over time, interpretation becomes difficult[[9](#_ENREF_9)]. If the investigator wants to use GEE to study time-varying predictors and discern the effects more clearly, he/she will have to separately specify the effects, both between-individual and within-individual, using additional variables [[10](#_ENREF_10)].

In summary, the GEE is a population view model that is good for studying population level mean outcome trajectory over time. It does not offer the possibility of individual level inference. The GEE also has a potentially restrictive assumption regarding missing data that needs to be taken into consideration when choosing this method. This model should be used when the population view is desired, e.g. healthcare utilization.

**2) MIXED EFFECTS REGRESSION MODEL (MRM)**

The MRM has many synonyms: random effects, multilevel model, hierarchical-linear-model[[11](#_ENREF_11), [12](#_ENREF_12)]. It has been used extensively in fields such as psychology, behavioural sciences and criminology. In contrast to the GEE, the MRM is subject-specific and models each individual’s outcome trajectory.

In MRM, each subject has a subject-specific outcome trajectory. All subjects’ trajectories are summarized into an average population trajectory. The equation below illustrates a simple case of a linear trajectory for an individual:

| Y_it_ | =(β_0_ + b_0i_) + | (β_1_ + b_1i_)time _it_ + | ε_it_ |
| --- | --- | --- | --- |
| Outcome Y for person i at time t | Intercept for person i | Slope for person i | Random error for person i at time t |

In the equation above, β_0_ represents the mean population intercept and β_1_ the mean population slope. Each individual i’s trajectory is related to the population intercept and slope (or whichever forms the time function takes) through their random effects b_0i_ and b_1i_ respectively. The random effects are unique person-specific characteristics that contribute to variability in outcome trajectories within a cohort. The random effect represents a combination of measured and unmeasured factors, e.g. genetic factors, environmental factors, prognostic factors and confounders[[10](#_ENREF_10)]. The final term in the equation ε_it_ represents the random error on each occasion of measurement, such that the actual values of the outcome measure would vary around each individual’s mean outcome trajectory.

Both the GEE and the MRM produce a population trajectory. While the mean trajectory in GEE is a curve of averages, the mean trajectory in MRM is an average of curves. As alluded to in the previous section, the mean outcome trajectory in GEE cannot necessarily be inferred to resemble an individual’s trajectory, especially in the cases of non-continuous outcomes. In the case of MRM, the individual’s outcome trajectory can be inferred from the population average outcome trajectory and is individualized by that individual’s random (b_0i_, b_1i_) and fixed effects.

The MRM allows data to be MAR[[7](#_ENREF_7), [13](#_ENREF_13)]. This is a much less restrictive assumption than the MCAR assumption for GEE. This kind of missing data is probably also more common in real-life clinic-based cohorts and therefore a more realistic assumption.

Both time-invariant and time-varying predictors can also be evaluated in the MRM. The time-invariant first encounter baseline DASm (bDAS) is an example of a between-individual prognostic factor that serves to distinguish patients’ responses. Time-invariant predictors associate with the intercept and predict the slope of disease trajectory. This kind of baseline factor may potentially be used to select different management strategies for individual patients who have been predicted to follow certain trajectories. A time-varying factor is an example of a within-individual prognostic factor that predicts within-individual change over time. This kind of factor may be targeted to improve the trend of the outcome trajectory within an individual over time.

As mentioned earlier, within-individual observations are usually correlated. Failure to adjust or misspecifying the covariance structure may lead to misleading conclusions from this model. In addition to the common covariance structures available in GEE, the MRM also allows easy implementation of spatial covariance structures [[14](#_ENREF_14)]. These covariance structures were initially developed for geospatial modeling[[15](#_ENREF_15)]. Spatial covariance structures are suitable when visits were not the result of a pre-planned schedule. When used in longitudinal models, the “distances” in spatial covariance structures are treated as time intervals.

In summary, the MRM is a subject view model that is potentially helpful in individual level inference of disease trajectory over time. One can say that an individual with a certain prognostic factor may follow a trajectory like so. In addition, it also offers a population view through the average population trajectory. The MRM can accommodate irregular and individualized visit schedules. This model should be used when the more refined individual view is preferred, such as the physician trying to refine management strategy according to patient characteristics or seeking to inform patients regarding prognosis.

**Additional model information in applying the GEE & MRM**

For the GEE model, we modeled within-individual correlations using a first order autoregressive structure (AR(1)). For the MRM, we modeled within-individual correlations with a spatial exponential covariance structure to reflect the individualized visit schedules in this observational cohort [[14](#_ENREF_14)]. For the MRM, our model posits natural heterogeneity i.e. random effects, in the intercept and the slope.

**3) LATENT CLASS TRAJECTORY ANALYSIS (LCTA)**

As rheumatologists, we have often observed that patients with similar diagnoses follow different courses and have different outcomes. Within the same disease cohort, patients may cluster into smaller subclasses in terms of disease evolution. Researchers have traditionally applied arbitrary criteria to classify patients into groups, e.g. observed disease activity at certain defined times[[16](#_ENREF_16), [17](#_ENREF_17)]. This approach runs the risk of being a self-fulfilling prophesy: there can only be as many groups as defined by the researchers and the outcome groups can only behave as defined by the investigators. It is more scientific to use a data-driven, statistical approach to identify these groups of patients with different prognoses. Furthermore, the use of a formal statistical structure also helps to distinguish random variations between individuals from real and systematically different variations.

Studies in social sciences and psychology have used latent class analysis to identify unobserved (i.e. latent) subgroups within a population, e.g. identifying latent classes of high-risk behaviors among adolescents[[18](#_ENREF_18)]. In much the same way, outcome trajectories of patients may cluster into a few more homogenous classes over time. Latent class trajectory analysis (LCTA) is the application of latent class analysis technique to longitudinal data, such that identifiable latent classes of (longitudinal) trajectories can be distinguished within the study population [[19](#_ENREF_19)]. Combined with baseline or early prognostic factors that predict an individual’s membership in a certain class of outcome trajectory, we can potentially use this information to refine patient management.

There are two main methods within the group of latent class trajectory analyses: group-based trajectory modeling [[20](#_ENREF_20), [21](#_ENREF_21)] and growth mixture modeling (GMM) [[22](#_ENREF_22)]. The GMM has gained great usage in literature. GMM identifies subclasses with distinct mean trajectories. Individuals’ trajectories within a subclass cluster around the mean class trajectory, deviating from it by their random effects, i.e. each class trajectory is defined by a class-specific MRM. GMM has been used to study diverse topics such as the development of bladder control in children, the prediction of treatment response among patients with interstitial cystitis, patterns of alcohol use, the evolution of aggressive behavior, the evolution of attention deficit/hyperactivity disorder, patterns of post myocardial infarction depression and patterns of fetal growth [[23-28](#_ENREF_23)]. A few recent papers in osteoarthritis have also used GMM to predict disease course [[29](#_ENREF_29), [30](#_ENREF_30)]. As such, we chose to demonstrate the application of GMM in this paper.

How can we use this class of model? We can ask if there are identifiable subclasses of outcome trajectories within a heterogeneous population of patients. The GMM is very flexible. Multiple outcome trajectories of disparate nature can be studied simultaneously (like the joint model in the previous section) [[24-26](#_ENREF_24), [31](#_ENREF_31)]. Furthermore, subclasses of an outcome can also take on trajectories of different shapes, e.g., some may have a linear shape while others have a quadratic shape.

In the MRM, we modeled subject specific trajectories and averaged them into an average population trajectory. The assumption in MRM is that all patients belong in the same population. In LCTA, we recognize that there may be more than one subgroup within the population and more than 1 kind of disease trajectory. Using LCTA, we can split a heterogeneous population of patients into several subgroups, each with its own subgroup specific average population trajectory. Each subgroup’s average population trajectory is an average of all individuals’ trajectories within that subgroup. This is akin to fitting multiple MRMs in one analysis. The subgroups can have different shapes of trajectories, thus informing on how one disease evolves differently in the different subgroups. The LCTA therefore has similar potential for individual inference as MRM.

After we identify distinct subclasses, we will then proceed to identify the prognostic factor(s) that predicts membership in the subclasses, i.e., membership predictors. When subclasses are robustly identified, the membership predictors will be able to clearly assign individuals into specific subclasses (i.e. with clear high probability in one of the subclasses).

Membership predictors are often time-invariant predictors. There are several ways of dealing with time-varying covariates in the context of growth mixture modeling. They can be studied as modifiers of disease outcome trajectories, with the assumption of a common effect across all subgroups or within subgroups of trajectories (i.e. different effects between subgroups). They can be studied in a joint model with the outcome trajectory of interest[[32](#_ENREF_32)]. Recent extension of the method now allows the effects of time-varying covariates to be studied in a piecewise fashion across the entire observation period with dynamic class changing between periods of observation[[33](#_ENREF_33)]. The specific formulation for time-varying covariates depends on the underlying research question and theoretical framework of the area of study. The interpretation of time-varying covariates in some of these formulations can be potentially difficult and complex. This is an area awaiting more work.

As the underlying model of LCTA is MRM, the assumption of missingness follows that of MRM, i.e. MAR. Furthermore, as in joint modeling, the LCTA has also been studied as a means of exploring for the effects of data missing not at random (MNAR)[[13](#_ENREF_13), [34](#_ENREF_34)].

In summary, researchers can use the LCTA to ask if there are more homogenous subgroups in a heterogeneous population of patients. They can ask about the shapes of multiple outcome longitudinal trajectories, e.g. SDASm and MDASm, which may together form a more global construct of outcome. Different kinds of outcomes e.g. continuous and binary, can be studied simultaneously, like in joint modeling. Identified membership predictors can classify individuals into more homogenous subgroups. Physicians can then use such predictors to individualize patient management according to their predicted prognostic trajectories.

**LCTA application to JDM cohort**

In the past, the LCTA (GMM) had to be performed using special software (MPlus) specifically developed for this analysis (http://www.statmodel.com). Now, common statistical software such as R, SAS (proc Traj) and Stata will also perform such an analysis [[35](#_ENREF_35)]. Another alternative is to cast this analysis in a Bayesian framework and then perform the analysis in a Bayesian software, e.g. WinBUGS ([www.mrc-bsu.cam.ac.uk/software/bugs](http://www.mrc-bsu.cam.ac.uk/software/bugs)). We performed this analysis using the package lcmm (v1.6.3) in R[[35](#_ENREF_35)].

We modeled trajectories using fractional polynomials with random intercepts and random slopes. We tested 2 to 5 latent classes for fit, using Bayesian Information Criteria (BIC) for comparisons. The 3-class, random slopes model fitted the data the best and most parsimoniously. We then went on to model the correlation between measurements, testing the 2 covariance structures available in the package: Brownian motion (BM) and Autoregressive (AR). The initial models using number of months since diagnosis as the basic time metric had difficulties converging, therefore we switched to using number of years (since diagnosis) as the basic time metric. The AR (time in years) models fitted significantly better than the BM models. We tested the AR model using multiple sets of starting values (>10), taking care to stay away from parameter boundaries, to ensure true estimate of the global maximum in parameter estimates.

From our analyses, 3 subclasses (AR corrected) appeared to be the best fit of our cohort. All the patients were classified clearly into 1 of the 3 classes, i.e. no patient was classified into 2 classes with similar probabilities. The 3 classes did not overlap significantly (see table 1). The proportions in the 3 classes were: 42%, 55% 3%. The last class only contained 3 patients. Future studies in another cohort of JDM patients will be helpful to validate the number of latent classes identified in this analysis.

**Table A2: Mean posterior probabilities of belonging in the 3 classes**

|  | **Probability in class 1** | **Probability in class 2** | **Probability in class 3** |
| --- | --- | --- | --- |
| **Class 1** | 0.9292 | 0.0689 | 0.0002 |
| **Class 2** | 0.0776 | 0.9214 | 0.0010 |
| **Class 3** | 0.0001 | 0.0243 | 0.9756 |

*Those in each of the 3 classes had correspondingly much lower probabilities of belonging in other classes, suggesting good separation of classes.*

**4) JOINT MODELING**

In monitoring a patient’s disease course, it is common to have more than one outcome measure of interest. The researcher may find interest in studying the shape of outcome trajectories, i.e. how outcomes evolve over time. We can ask whether and how two or more outcomes are related in their evolutions over time, e,g., lupus disease activity (SLEDAI) [[36](#_ENREF_36)] and depression scores. Joint modeling allows us to test for concordance or discordance in the ways that the outcome trajectories evolve over time. The joint outcomes of interest do not have to be of a similar nature; they can be of any combination, e.g. a continuous with a binary outcome, a continuous with a time-to-event outcome and any number of outcomes. Joint modeling has also been used as a means of reducing bias when there is informative attrition (e.g. when patients drop out because they are very well or very sick)[[7](#_ENREF_7), [37](#_ENREF_37), [38](#_ENREF_38)]. Joint modeling of the outcome of interest with the time-to-dropout has been used to address this problem of informative attrition. Joint modeling is thus a potentially powerful tool that can expand our understanding of how outcomes trajectories evolve over time.

As we have demonstrated in the preceding sections, longitudinal data can be studied at a marginal level (e.g. GEE) or an individual level (e.g. MRM), so can joint models be formulated in either form, depending on the underlying research questions. Due to the assumptions required of joint marginal models, these are not very flexible[[8](#_ENREF_8)]. The MRM formulation is more flexible and can be easily extended to outcomes of disparate nature (e.g. continuous, binary or count). As such, the MRM formulation is more commonly used. For the MRM formulation, it is assumed that the outcomes are independent, conditional on the random effects, i.e. the underlying characteristics of the individual (random effects) govern the outcomes[[39-41](#_ENREF_39)]. The longitudinal process can also be jointly modeled with a time-to-event outcome using a proportional hazards model. Newer literature does not even require that random effects be shared between outcomes now[[32](#_ENREF_32), [33](#_ENREF_33)]. Instead, it is assumed that the outcome trajectories are manifestations of a common latent process[[32](#_ENREF_32)].

Missing data is allowed in the joint model. The mechanism of missing data depends on the underlying model used: marginal (GEE) or subject-specific (MRM), used in specifying the joint model. If the investigator chooses to use the GEE, then missing data is assumed to be MCAR. If the investigator chooses the more common choice of MRM, then missing data is assumed to be MAR. A special form of the joint model– the joint model of random effects– where the longitudinal trajectory of interest is jointly modeled with the time-to-dropout, has been proposed as a means of exploring the impact of MNAR, i.e., informative attrition[[8](#_ENREF_8), [13](#_ENREF_13), [39](#_ENREF_39)]. When data is MNAR, the missing outcome is related to the missing process[[13](#_ENREF_13)]. An example of this would be the sicker patients who no longer return for follow-up as they are too sick to leave their home. This kind of missing data can potentially bias any inference made based on patients remaining in the cohort. The joint model can be used to explore and adjust for this kind of bias [[37](#_ENREF_37), [39](#_ENREF_39)]. This does not mean that all the bias of MNAR can be resolved, as such modeling tends to lean on many assumptions, some of which may not be possible to verify.

The joint model allows for testing of both time-invariant and time-varying predictors with similar interpretations as in the MRM. Within the same model, one can study the relative effects (in both the significance and the effect size) of different predictors on each of the outcome trajectories modeled.

These models have been used in a few areas of medicine. Multiple continuous outcomes, e.g., hearing at various frequencies in both ears, have been jointly modeled to evaluate the change in hearing ability as patients age and to identify factors influencing those changes [[41](#_ENREF_41)]. A continuous (sequential organ failure assessment score) outcome has been jointly modeled with discharge status from intensive care unit (ICU) to evaluate the effect of treatment on outcome trajectory, adjusted by the dropout process, through either death or discharge from the ICU [[42](#_ENREF_42)].

This class of models has not yet been commonly used in the rheumatology literature. We believe that it has a lot of potential in answering real-life clinical questions.

In summary, the researcher should consider joint modeling when seeking to study multiple outcome trajectories concurrently. The multivariate analyses may be more accurate reflections of the clinical decision making process, whereby several outcomes are followed and all factored into the clinician’s global perception of a patient’s outcome. The researcher can also investigate the degree of concordance among outcome trajectories. In addition, the researcher can potentially use this kind of model to explore for the biasing effects of missing data on observed outcomes.

**Joint Modeling applied to JDM outcomes**

This joint model was specified using the MRM. We used the spatial exponential covariance structure for modeling the MDASm and SDASm trajectories, similar to the section on MRM. The MDAS was modeled with a 2^nd^ order fractional polynomial (m=2, p= –1,–2). The SDAS was also modeled with a 2^nd^ order fractional polynomial but of a different form (m=2, p= –1,–1). Both forms were selected based on the maximal gain in fit[[1](#_ENREF_1)]. When modelling for predictors, we crossed the fractional polynomial time forms of the MDAS and SDAS curves with bDAS to test the effects of bDAS on the slopes. Only the one time term of MDAS (p= –1, which is in common with that of the SDAS) was significant, so this was retained in the model. We left the product of the bDAS crossed with first time term of SDAS in the equation for easier comparison but this product was not significant, ie. bDAS did not predict the slope evolution of SDAS.

**Summary**

Modern longitudinal analytic methods are now more flexible and powerful. Observational studies, with challenges from irregular visit schedules and missing data, can now be analyzed using these longitudinal methods. The choice of method will depend on the researcher’s question. This review is not meant to be exhaustive. Markov multistate models[[43](#_ENREF_43)] or latent transition regression[[44](#_ENREF_44)] are considered longitudinal as well but as they do not deal with “trajectories” but a change in state, we did not include them in this review.

**References**

1. Royston P, Altman DG: Regression using fractional polynomials of continuous covariates: parsimonious parametric modelling. *Applied Statistics* 1994, 43**:**429-467.

2. Sauerbrei W, Royston P: Building multivariable prognostic and diagnostic models: transformation of the predictors by using fractional polynomials. *Journal of the Royal Statistical Society: Series A* 1999, 162**:**71-94.

3. Zeger SL, Liang KY, Albert PS: Models for longitudinal data: a generalized estimating equation approach. *Biometrics* 1988, 44**:**1049-1060.

4. Lim LS, Lee SJ, Feldman BM, Gladman DD, Pullenayegum E, Uleryk E, Silverman ED: **A systematic review of the quality of prognosis studies in systemic lupus erythematosus.** In *Arthritis care & research*, 2014/03/20 edition; 2014.

5. Lin H, Scharfstein DO, Rosenheck RA: Analysis of longitudinal data with irregular, outcome-dependent follow-up. *Journal of the Royal Statistical Society: Series B (Statistical Methodology)* 2004, 66**:**791-813.

6. Preisser JS, Lohman KK, Rathouz PJ: Performance of Weighted Estimating Equations for Longitudinal Binary Data with Drop-Outs Missing at Random. *Statistics in Medicine* 2002, 21.

7. Rubin DB: Inference and missing data. *Biometrika* 1976, 63**:**581-592.

8. Fitzmaurice G, Davidian M, Verbeke G, Molenberghs G (Eds.): **Longitudinal Data Analysis**. Boca Raton: Chapman & Hall/CRC Press; 2009.

9. Twisk JWR: *Applied Longitudinal Data Analysis for Epidemiology. A Practical Guide.* New York: Cambridge University Press; 2003.

10. Diggle P, Heagerty P, Liang KY, Zeger S: *Analysis of Longitudinal Data.* 2nd edn. Oxford: Oxford University Press; 2002.

11. bryk a, raudenbush s: Application of hierarchial linear models to assessing change. . *Psychol Bull* 1987, 101**:**147-158.

12. Singer JD, Willett JB: *Applied Longitudinal Data Analysis- Modeling Change and Event Occurrence.* New York: Oxford University Press

; 2003.

13. Sterne JA, White IR, Carlin JB, Spratt M, Royston P, Kenward MG, Wood AM, Carpenter JR: Multiple imputation for missing data in epidemiological and clinical research: potential and pitfalls. *BMJ* 2009, 338**:**b2393.

14. Moser EB: **Repeated Measures Modeling with PROC MIXED.** In *SAS Users Global International (SUGI); Montreal, Canada*. SAS; 2004: Paper 188-129.

15. Singh VP, Fiorentino M (Eds.): **Water Science and Technology Library**. The Netherlands: Kluwer Academic Publishers; 1996.

16. Nikpour M, Gladman DD, Ibanez D, Harvey PJ, Urowitz MB: Variability over time and correlates of cholesterol and blood pressure in systemic lupus erythematosus: a longitudinal cohort study. *Arthritis Research & Therapy*, 12**:**R125.

17. Stringer E, Singh-Grewal D, Feldman BM: Predicting the course of juvenile dermatomyositis: significance of early clinical and laboratory features.[Erratum appears in Arthritis Rheum. 2008 Dec;58(12):3950]. *Arthritis & Rheumatism* 2008, 58**:**3585-3592.

18. Collins LM, Lanza ST: *Latent class and latent transition analysis: With applications in the social, behavioural, and health sciences.* New York: Wiley; 2010.

19. Nagin DS, Odgers CL: Group-based trajectory modeling in clinical research. *Annu Rev Clin Psychol*, 6**:**109-138.

20. Nagin D: *Group based modeling of development.* Cambridge, Massachusetts: Harvard University Press; 2005.

21. Nagin D: Analyzing developmental trajectories: a semiparametric, group-based approach. *Psychol Methods* 1999, 4**:**139-157.

22. Muthen B, Shedden K: Finite mixture modeling with mixture outcomes using the EM algorithm. *Biometrics* 1999, 55**:**463-469.

23. Kerner B, Muthen BO: Growth mixture modelling in families of the Framingham Heart Study. *BMC Proc* 2009, 3 Suppl 7**:**S114.

24. Elliott MR, Gallo JJ, Ten Have TR, Bogner HR, Katz IR: Using a Bayesian latent growth curve model to identify trajectories of positive affect and negative events following myocardial infarction. *Biostatistics* 2005, 6**:**119-143.

25. Leiby BE: Identification of multivariate responders and non-responders by using Bayesian growth curve latent class models. *Appl Statist* 2009, 2009**:**505-524.

26. Neelon B, Swamy G, K. , Burgette LF, Miranda M: A Bayesian growth mixture model to examine maternal hypertension and birth outcomes. *Stat Med* 2011.

27. Neelon BH, O'Malley AJ, Normand SL: A Bayesian model for repeated measures zero-inflated count data with application to outpatient psychiatric service use. *Statistical modelling* 2010, 10**:**421-439.

28. Slaughter JC, Herring AH, Thorp JM: A Bayesian latent variable mixture model for longitudinal fetal growth. *Biometrics* 2009, 65**:**1233-1242.

29. Holla JF, van der Leeden M, Heymans MW, Roorda LD, Bierma-Zeinstra SM, Boers M, Lems WF, Steultjens MP, Dekker J: Three trajectories of activity limitations in early symptomatic knee osteoarthritis: a 5-year follow-up study. *Annals of the rheumatic diseases* 2013.

30. Verkleij SP, Hoekstra T, Rozendaal RM, Waarsing JH, Koes BW, Luijsterburg PA, Bierma-Zeinstra SM: Defining discriminative pain trajectories in hip osteoarthritis over a 2-year time period. *Annals of the rheumatic diseases* 2012, 71**:**1517-1523.

31. Neelon BH, O'Malley AJ, Normand SL: A Bayesian model for repeated measures zero-inflated count data with application to outpatient psychiatric service use. *Stat Modelling*, 10**:**421-439.

32. Proust-Lima C, Letenneur L, Jacqmin-Gadda H: A nonlinear latent class model for joint analysis of multivariate longitudinal data and a binary outcome. *Stat Med* 2007, 2007**:**2229-2245.

33. Lin H, Han L, Peduzzi PN, Murphy TE, Gill TM, Allore HG: A dynamic trajectory class model for intensive longitudinal categorical outcome. *Statistics in medicine* 2014, 33**:**2645-2664.

34. Muthen B, Asparouhov T, Hunter AM, Leuchter AF: Growth modeling with nonignorable dropout: alternative analyses of the STAR*D antidepressant trial. *Psychological methods* 2011, 16**:**17-33.

35. **Package 'lcmm'** [cran.r-project.org/web/packages/lcmm/lcmm.pdf‎]

36. Bombardier C, Gladman DD, Urowitz MB, Caron D, Chang CH: Derivation of the SLEDAI. A disease activity index for lupus patients. The Committee on Prognosis Studies in SLE. *Arthritis & Rheumatism* 1992, 35**:**630-640.

37. Hogan JW, Laird NM: Increasing efficiency from censored survival data by using random effects to model longitudinal covariates. *Stat Methods Med Res* 1998, 7**:**28-48.

38. Lim E, Ali A, Theodorou P, Sousa I, Ashrafian H, Chamageorgakis T, Duncan A, Henein M, Diggle P, Pepper J: Longitudinal study of the profile and predictors of left ventricular mass regression after stentless aortic valve replacement. *The Annals of thoracic surgery* 2008, 85**:**2026-2029.

39. Diggle PJ, Sousa I, Chetwynd AG: Joint modelling of repeated measurements and time-to-event outcomes: the fourth Armitage lecture. *Stat Med* 2008, 27**:**2981-2998.

40. Fieuws S, Verbeke G: Joint modelling of multivariate longitudinal profiles: pitfalls of the random-effects approach. *Statistics in medicine* 2004, 23**:**3093-3104.

41. Fieuws S, Verbeke G: Pairwise fitting of mixed models for the joint modeling of multivariate longitudinal profiles. *Biometrics* 2006, 62**:**424-431.

42. Deslandes E, Chevret S: Joint modeling of multivariate longitudinal data and the dropout process in a competing risk setting: application to ICU data. *BMC medical research methodology* 2010, 10**:**69.

43. Commenges D: Multi-state models in epidemiology. *Lifetime Data Analysis* 1999, 5**:**315-327.

44. Miglioretti DL: Latent transition regression for mixed outcomes. *Biometrics* 2003, 59**:**710-720.
